# Complete Genome Sequence of *Escherichia coli* ER1821R, a Laboratory K-12 Derivative Engineered To Be Deficient in All Methylcytosine and Methyladenine Restriction Systems

**DOI:** 10.1128/genomeA.00763-16

**Published:** 2016-08-11

**Authors:** Michael G. Jobling, Elisabeth A. Raleigh, Daniel N. Frank

**Affiliations:** a University of Colorado Anschutz Medical Campus, Aurora, Colorado, USA; b New England Biolabs, Ipswich, Massachusetts, USA

## Abstract

We present here the complete genomic sequence of a rifampin-resistant derivative of the *Escherichia coli* K-12 laboratory strain ER1821, engineered to be deficient in all known restriction systems, making it suitable for generating unbiased libraries from organisms with non-K-12 methylation patterns. The ER1821R genome is most closely related to that of DH1, another popular cloning strain (both derived from MM294), but is deleted for the *e14* prophage (McrA^-^) and the immigration control (McrBC^-^ EcoKI R^-^ M^-^ Mrr^-^) loci.

## GENOME ANNOUNCEMENT

Wild-type *Escherichia coli* strains restrict incoming DNA with foreign patterns of nucleotide modification (1, 2), reducing the recovery of gene libraries containing such modifications. *E. coli* K-12 derivative ER1821, engineered to be restriction deficient (3), has been useful for library preparation (4) and cloning DNA methyltransferases (5, 6). ER1821 is derived from MM294 (7), from which the popular cloning strain DH1 (MM294 *recA gyrA*) was also derived (8). The restriction systems were removed using P1 transduction to cure the *e14* prophage encoding *mcrA* and delete the immigration control region (ICR) Δ(*mcrC-hsd-mrr*)*114*::IS*10* (9). An Hfr mating transferred *recA* to MM294 to form DH1; a large segment of flanking DNA likely accompanied the selected allele. The precise limits of these regions are unknown, and a completed genome for a different MM294 descendant will permit comparison and be beneficial to the scientific community.

Total DNA from a single transconjugant of a rifampin-resistant derivative of ER1821 carrying a *Salmonella* ceftriaxone resistance plasmid was used to determine the plasmid genome, which will be described elsewhere. A genomic library, constructed using an Illumina Nextera XT kit, produced 1.76 × 10^6^ 300-bp paired-end reads on a MiSeq instrument. A *de novo* assembly by the Geneious assembler (version 6.1.7) (10) produced 84 contigs >1 kb (*N*_50_, 103 kb). The full data set, mapped to the DH1 genome (accession no. NC_017625.1) using the Geneious Read Mapper, produced a consensus sequence with 90-fold mean coverage, two large gaps corresponding to the *e14* and ICR deletions, and six discordant regions. Complete sequences covering these regions were extracted from the *de novo* assembly, and the two deletions, five IS insertions, a small expansion of REP161, and a phase-variable inversion switching *fimA* to “on” (11) were incorporated into the consensus sequence, producing a complete circular genome of 4,595,577 bp. The full data set remapped to this assembly with no gaps or discordant regions. ER1821R and DH1 show 54 single nucleotide polymorphisms (SNPs), excluding insertions and deletions. Thirty-six SNPs in an 842-kb segment of DH1 surround *recA* from the Hfr donor. Here, ER1821R has *rpoS396*(am) and *luxS*^+^ (wild-type [WT] and frameshift in DH1) and a novel IS*3* insertion into *lrhA*, a negative regulator of motility (11). The *e14* prophage is precisely deleted but is closely linked to three novel IS insertions (IS*10*, IS*1R*, and IS*2*). All but one (*purB*20 [12]) of the remaining SNPs are in 65 kb surrounding the 24-kb ICR deletion, replaced by a single IS*10*.

ER1821R is *relA*^+^ *spoT*^+^ but has ancestral *rfbD1* and *creC510* mutations. Finally, ER1821R has an *rpoB* mutation conferring rifampin resistance (13). Thus, the genotype for ER1821 becomes λ^-^ F^-^ *glnX44 e14^-^* (McrA*^-^*) *rfbD1 endA1 thi-1* Δ(*yjiT-opgB*)*114*::IS*10* (EcoKI R^-^ M^-^ McrBC^-^ Mrr^-^) + *rpoS393*(am) *creC510 lrhA*::IS*3 ydeN*::IS*10*. These newly detected mutations highlight the inherent problems using matings, transduction, and active transposons to transfer markers, which can introduce additional mutations. Comparing ER1821R with DH1, we can infer the likely sequence of the MM294 parent to be ER1821-like through 2 Mb (but *lrhA*^+^) and DH1-like (but *purB*^+^ *gyrA*^+^) for the remainder.

### Accession number(s).

The complete genomic sequence of ER1821R (annotated by the NCBI’s prokaryotic genomic pipeline (http://www.ncbi.nlm.nih.gov/genome/annotation_prok/) is available in GenBank with accession no. CP016018.
